# Low-dose overlap initiation with split tablets of buprenorphine in intubated intensive care unit patients with opioid use disorder

**DOI:** 10.1186/s12954-024-01028-4

**Published:** 2024-06-07

**Authors:** Laura Szczesniak, Sarah Britton, Theresa Baxter RN, Ross Sullivan

**Affiliations:** 1 Department of Emergency Medicine, Medical College of Wisconsin, Milwaukee, WI 53208 USA; 2 Present Address: Department of Clinical Pharmacy, Syracuse VA Medical Center, Syracuse, NY 13210 USA; 3 Department of Addiction Medicine, Upstate Medical University, Syracuse, NY 13210 USA; 4 Department of Emergency Medicine, Upstate Medical University, Syracuse, NY 13210 USA; 5 Department of Pharmacy, Upstate Medical University, Syracuse, NY, 13210, USA

**Keywords:** Buprenorphine, Opioid use disorder, Medications to treat opioid use disorder, Withdrawal

## Abstract

**Background:**

As the opioid public health crisis evolves to include fentanyl and other potent synthetic opioids, more patients are admitted to the hospital with serious complications of drug use and frequently require higher levels of care, including intensive care unit (ICU) admission, for acute and chronic conditions related to opioid use disorder (OUD). This patient population poses a unique challenge when managing sedation and ensuring adequate ventilation while intubated given their high opioid requirements. Starting a patient on medications such as buprenorphine may be difficult for inpatient providers unfamiliar with its use, which may lead to undertreatment of patients with OUD, prolonged mechanical ventilation and length of stay.

**Methods:**

We developed a 7-day buprenorphine low dose overlap initiation (LDOI) schedule for patients with OUD admitted to the ICU (Table 1). Buprenorphine tablets were split by pharmacists and placed into pre-made blister packs as a kit to be loaded into the automated medication dispensing machine for nursing to administer daily. An internal quality review validated the appropriate dosing of split-dose tablets. To simplify order entry and increase prescriber comfort with this new protocol, we generated an order set within our electronic health record software with prebuilt buprenorphine titration orders. This protocol was implemented alongside patient and healthcare team education and counseling on the LDOI process, with follow-up offered to all patients upon discharge.

**Results:**

Here we report a series of 6 ICU patients started on buprenorphine using the LDOI schedule with split buprenorphine tablets. None of the 6 patients experienced precipitated withdrawal upon buprenorphine initiation using the LDOI schedule, and 5/6 patients were successfully extubated during the buprenorphine initiation. Four of six patients had a decrease in daily morphine milligram equivalents, with 3 patients transitioning to buprenorphine alone.

**Conclusion:**

Initiating buprenorphine via LDOI was found to be successful in the development of a protocol for critically ill patients with OUD. We examined LDOI of buprenorphine in intubated ICU patients and found no events of acute precipitated withdrawal. This protocol can be used as a guide for other institutions seeking to start critically ill patients on medication treatment for OUD during ICU admission.

## Background

In the United States, opioids were involved in over 80,411 overdose deaths in 2021 alone, representing over 75% of drug overdose deaths [1]. Patients with opioid use disorder (OUD) often have a significantly longer length of stay when hospitalized [2], and more post-operative complications when compared to patient populations without a history of OUD [3, 4]. Substance use disorder can also contribute to a patient leaving a hospital early against medical advice [5]. In a critical care setting, patients with OUD are more likely to die from COVID-19 [6, 7], have a greater length of stay and ultimately greater healthcare costs [2]. Managing patients with OUD is becoming even more challenging as fentanyl and other synthetic opioids become increasingly prevalent and opioid tolerance continues to increase [8].

Buprenorphine is a partial opioid mu-agonist and is a medication to treat opioid use disorder (MOUD). Buprenorphine is notable for its reduced overdose potential and relative safety, which has led to an enormous growth in buprenorphine prescribing for OUD [9]. Treatment with buprenorphine has slowly expanded from outpatient to within the hospital setting, including in the emergency department [10] and inpatient [11], but there is relatively little data on patients admitted to hospital intensive care units (ICUs) or on ventilators. Given the significant challenge of balancing sedation, ventilator compliance, pain management and treatment of OUD, the use of MOUD in critically ill patients can prove challenging.

A recent case report demonstrated the potential to start buprenorphine with concurrent fentanyl infusion in the ICU [12]. Given that patients with OUD suffer increased morbidity and mortality compared to patients without substance use disorders, it is paramount that providers identify areas to improve patient care in this population to minimize further harm from OUD.

In the current era of synthetic opioids with increasingly high potency, the authors note that opioid tolerance has become more prevalent in ICU patients within our institution. Anecdotally, agitation secondary to opioid withdrawal in these patients has resulted in patients being placed on high dose fentanyl infusions, delays in extubation, and in many instances, re-intubation. In order to mitigate complications related to sedation and ventilation in hospitalized patients with OUD, we developed a protocol for initiation of buprenorphine on intubated patients in the ICU. Here we report a series of 6 patients with OUD who underwent low-dose overlap initiation (LDOI) with split tablet buprenorphine while intubated in the ICU. To the best of the authors’ knowledge, this is the first use of split tablet buprenorphine for LDOI in ICU patients while intubated.

## Methods

### Setting and context

The protocol was developed in an urban tertiary care public hospital on the east coast with 752 total inpatient beds across 2 campuses. There are approximately 120 ICU beds with > 80% being Medical ICU’s. The hospitals serve an ethnically, racially, and geographically diverse population.

An Addiction Medicine service practices within the hospitals, serving a consultative role. The Addiction Medicine service is comprised of a board-certified Addiction Medicine physician, a board-certified Addiction Medicine advanced practice provider, rotating Medical Toxicology fellows, Psychiatry residents and/or fellows, and rotating advanced practice providers. The Addiction Medicine consultative service typically rounds on 10–20 patients per day and is available for consultation on a continuous basis, including overnight and on weekends.

### Establishment of buprenorphine low dose overlap initiation protocol

We developed a 7-day buprenorphine LDOI schedule as outlined in Table 1. Patients remain on opioid agonists (intravenous fentanyl infusion) upon buprenorphine initiation. Initially, on days 1–3 of the regimen, the patient receives very low doses of buprenorphine. To provide such doses, the hospital pharmacy uses buprenorphine 2 mg manufactured unit dose tablets, which are pre-packed as split tabs containing partial doses (one quarter of a 2 mg tablet being a dose of approximately 0.5 mg). Split tabs are available in automated medication dispensing machine (Pyxis^™^ MedStation^™^) on medical floors. Starting on day 4 of the LDOI regimen, buprenorphine split doses are no longer required. The buprenorphine target dose of 16 mg/day is reached on day 7 of the protocol, which is when discontinuing or tapering other opioid agonists, including fentanyl, is recommended based on individual patient condition. The buprenorphine is then continued at the target dose per specific patient, with follow-up offered to all patients upon discharge.

**Table 1:** Buprenorphine low dose overlap initiation (LDOI) schedule

Day	Daily buprenorphine dosing sublingual (mg)	Number of tablet(s) per dose*	Total Daily Dose of buprenorphine (mg)	Pre-existing Opioid Agonist (e.g. fentanyl infusion)
1	0.5 mg QD	One-quarter tab	0.5 mg	Continue
2	0.5 mg BID	One-quarter tab	1 mg	Continue
3	1 mg BID	One-half tab	2 mg	Continue
4	2 mg BID	1 tab	4 mg	Continue
5	4 mg BID	1 tab	8 mg	Continue
6	4 mg TID	1 tab	12 mg	Continue
7+	8 mg BID	2 tabs^**+**^	16 mg	Stop/Taper

*QD = every day, BID = twice daily, TID = three times daily*

* buprenorphine tablets available in 2 mg or 4 mg dosage

^***+***^ *dose may be further increased based on individual patient circumstances*

An internal quality review of buprenorphine tablets is performed by one of the hospital’s pharmacists, who inspects the tablets to ensure that they are split evenly. Tablets that do not appear to have a clean split or showing signs of fragmentation are discarded.

### Implementation of low dose overlap initiation protocol

The protocol was implemented as a coordinated effort between the primary service (Critical Care Medicine), Pharmacy, and the Addiction Medicine consultative service. Patients were eligible for the LDOI protocol if they had a documented history of OUD, on mechanical ventilation and requiring an opioid infusion (fentanyl) for adequate sedation, with extubation expected during initiation of buprenorphine.

After a consult was placed, the Addiction Medicine service implemented the LDOI protocol as an order set within the electronic medical record (EMR, Epic^™^). Each day, nursing staff removed the pre-packaged buprenorphine dose from the automated medication dispensing machine for sublingual patient administration. The Addiction Medicine service visited patients at bedside on a daily basis to evaluate for any symptoms of precipitated withdrawal, including piloerection, tremors, vomiting, diarrhea, agitation, diaphoresis, and tachycardia [13], and provided ongoing recommendations to the primary teams throughout the process and for the duration of the patient’s hospital admission. Healthcare team education was performed, and patients were informed of their treatment with buprenorphine upon extubation. During this time, patients were also given the option to opt out of the LDOI protocol.

### Review of patient outcomes after LDOI protocol

A chart review was carried out on a total of 6 patient admitted to the ICU who underwent the buprenorphine LDOI protocol. A query of the EMR was performed to identify patient demographics, any documented symptoms of withdrawal, pre- and post-LDOI daily morphine milligram-equivalents (MME), as well as whether the patient was extubated during the LDOI protocol.

## Results

Six patients were reviewed after LDOI with buprenorphine (Table 2). All patients had received a diagnosis of OUD prior to buprenorphine initiation, had a history of intravenous drug use, and had been on fentanyl continuous infusions while on mechanical ventilation in the ICU. All patients except Patient 2 had received buprenorphine previously based on review of the Prescription Drug Monitoring Program. No symptoms of precipitated withdrawal were evident in any of the patients upon LDOI with buprenorphine. Five out of six patients were successfully extubated during the process and consented to continue buprenorphine after education on buprenorphine and the LDOI protocol. No patients reported symptoms of withdrawal upon extubation. These patients were prescribed buprenorphine on discharge with a follow-up Addiction clinic appointment scheduled. One patient expired during the LDOI (Patient 5) due to pre-existing complications. Pre- and post-LDOI daily MME ranged from 80 to 1800 and 0-1200, respectively. Four out of six patients decreased daily MME requirements, and three patients did not require any opioids aside from buprenorphine after LDOI.

**Table 2:** Patient demographics

Patient	Age	Sex	Race	Days opioid treatment pre-LDOI	Documented Precipitated Withdrawal?	Pre-LDOI daily MME	Post-LDOI daily MME*	Days intubated pre-LDOI	Extubated during LDOI?
1	33	M	Caucasian	8	No	1080	1200	8	Yes
2	23	M	Caucasian	6	No	1800	0	3	Yes
3	39	M	Caucasian	1	No	80	7.5	1	Yes
4	39	M	Caucasian	7	No	1500	0	7	Yes
5	35	F	Caucasian	2	No	720	720	1	No
6	35	F	African American	1	No	1140	0	1	Yes

* daily MME does not include buprenorphine dosing

## Discussion

Prior to the development of the LDOI protocol, the authors noted an increased frequency of Addiction Medicine consults for ICU patients with OUD. Patient agitation from opioid withdrawal was prolonging intubation or causing a need for re-intubation in these patients. The LDOI protocol was developed as a quality improvement project to address opioid debts in tolerant individuals and mitigate issues surrounding patient extubation.

Buprenorphine provides a means to manage high opioid requirements without causing significant respiratory depression and can reduce time to extubation by managing patient agitation secondary to opioid withdrawal. Our hospital successfully developed and deployed a LDOI regimen to provide optimal care to our patients suffering from opioid use disorder. The development of this LDOI regimen originated from a collaborative effort among providers and pharmacists. We found that both patient/family education as well as education of medical providers are crucial to successfully utilizing this protocol. Education focuses on the pharmacological reason for using LDOI, highlighting the rationale for administering low doses of buprenorphine on the initial protocol days and emphasizing the goal to minimize withdrawal symptoms.

LDOI regimens that utilize partial sublingual tablets have been described in literature [14–21]. Buprenorphine mono-product is preferred for our regimen because that is the product our pharmacy demonstrated as having the ability to cleanly split, and is readily available on the hospital formulary. Rather than splitting tablets as orders are placed, buprenorphine partial tablets are prepacked and dispensed as unit doses from the hospital pharmacy. Pre-packing split tablets allows for accurate nursing administration of the ordered dose without further manipulation. Additional benefits of pharmacy pre-packing include reduced opioid waste and accurate documentation requirements associated with administering partial doses of controlled substances.

Many current practices for LDOI involve cutting buprenorphine films into smaller pieces, which can be both tedious and imprecise for hospital staff; other practices include buccal or intravenous formulations that are not on this hospital’s formulary. Our current method of splitting buprenorphine tablets resulted in no adverse effects, no observable opioid withdrawal, and most importantly has allowed use of on-hand buprenorphine preparation. With this streamlined method, we hope to reduce hesitancy among providers caring for patients with OUD.

Initiating a buprenorphine LDOI regimen as individual buprenorphine orders can be prone to errors and time consuming. Therefore, we created an order set within our EHR that includes prebuilt buprenorphine titrating orders. The order set allows for simplified order entry and increased prescriber comfort with this new LDOI strategy. Additionally, within the order set, nurses are instructed to notify a provider if withdrawal symptoms occur as evidenced by signs and symptoms. With this being a new protocol at our hospital, the Addiction Medicine consultative service must either be the ordering provider or must make official recommendations as consultants to use the protocol.

Here we review 6 patients who underwent LDOI with buprenorphine as a proof-of-concept. Upon implementation of the protocol, 4/6 patients had a reduced daily MME post-LDOI (Table 2). It is important to note that buprenorphine was not converted to MME. For the purposes of this study, MME calculations serve more to show opioid requirements from sources aside from buprenorphine.

Development and implementation of the LDOI protocol does raise ethical questions regarding the appropriateness of starting a critically ill patient on MOUD, as all patients reviewed were intubated and sedated, and therefore unable to provide consent for treatment. The authors strongly believe that the buprenorphine LDOI protocol will serve as an effective method in harm reduction, particularly in this era of high opioid tolerance related to spikes in synthetic opioid use, as patients had been subject to significant morbidity (discomfort on the ventilator, prolonged intubation, re-intubation, high dose fentanyl drips). Furthermore, all patients were educated on the goals of minimizing withdrawal symptoms using the LDOI protocol on extubation and given the option to stop buprenorphine. Although we cannot prevent all collateral harm from this protocol, we will continue to improve its implementation in vulnerable patient populations.

This study has limitations. Given the retrospective nature of the analysis, this study was only observational and had no patient controls to compare outcomes, therefore no formal analysis was performed. Despite no objective signs of withdrawal in patients initiated on buprenorphine, patient sedation with opioid and non-opioid medications such as benzodiazepines precluded accurate tracking of a complete Clinical Opiate Withdrawal Scale [13] including subjective symptoms (myalgias, anxiety, restlessness) during the LDOI protocol. This study should serve more as proof-of-concept and results cannot be generalized to larger populations given the small patient population observed. Additionally, our institution benefits from having a dedicated Addiction Medicine consultative service to supervise and implement a buprenorphine LDOI regimen, which may be more difficult to implement in smaller hospitals with fewer consulting services.

## Conclusions

We have reported the successful development and implementation of a buprenorphine LDOI protocol in an ICU setting. Our small retrospective case series demonstrates that it is efficacious and safe to spilt buprenorphine tablets for administration to intubated ICU patients with OUD. There was no observable opioid withdrawal after dosing, and none of the patients reported symptoms of opioid withdrawal upon extubation. Further studies are warranted in the use of split dosing buprenorphine tablets for LDOI in all hospitalized patients.

## Data Availability

No datasets were generated or analysed during the current study.

## Abbreviations

COWS: Clinical Opiate Withdrawal Score
ICU: Intensive Care Unit
LDOI: Low Dose Overlap Initiation
MME: Morphine Milligram Equivalent
MOUD: Medications To Treat Opioid Use Disorder
OUD: Opioid Use Disorder
